# Performance of the ActiGraph accelerometer using a national population-based sample of youth and adults

**DOI:** 10.1186/s13104-014-0970-2

**Published:** 2015-01-17

**Authors:** Kelly R Evenson, Fang Wen

**Affiliations:** Department of Epidemiology, Gillings School of Global Public Health, University of North Carolina, 137 East Franklin Street, Suite 306, Chapel Hill, North Carolina 27514 USA

**Keywords:** ActiGraph, Intensity, Missingness, Non-wear, Physical activity, Sample weights, Sedentary behavior

## Abstract

**Background:**

Accelerometer output may be semi-continuous or continuous in nature, which has implications on discerning non-wear and defining physical activity intensity levels. This study described field-based accelerometer performance from a surveillance sample of youth and adults.

**Methods:**

Using 2003–2006 National Health and Nutrition Examination Survey data, 4,028 youth ages 6 to 17 years and 7,931 adults age > =18 years wore an ActiGraph AM7164 accelerometer for one week, providing at least 3 days of wear for > =8 hours/day. Accelerometer performance was assessed by exploring the number of different values of accelerometer counts/minute for each participant.

**Results:**

On average, youth participants had 1381 different counts/minute over 7 days (median 1360, interquartile range 1127–1623) and adult participants had 1101 different counts/minute over 7 days (median 1085, interquartile range 874–1313). For both youth and adults, when restricting to counts/minute between 0 to 4999, every possible value (in counts/minute) occurred at least once.

**Conclusion:**

The field-based data confirmed that the accelerometer used in this study allowed for continuous counts/minute through which all but the most vigorous activities would usually occur.

## Background

Accelerometers are wearable devices that measure acceleration or movement, allowing for estimation of physical activity and sedentary behavior. Currently, two of the most commonly used accelerometers in epidemiologic and surveillance studies are the ActiGraph and Actical. Both devices assess acceleration using counts as the output metric [1]. A recent study found that the Actical counts were semi-continuous in nature, with some values never registering, particularly at the low end of the spectrum where sedentary and light physical activity would occur [2].

There are several implications for this finding. The lower variability of the device in the sedentary range poses a greater challenge to researchers trying to distinguish whether zero counts/minute represents sedentary behavior or non-wear. The semi-continuous nature of the data may also contribute to inaccuracies in using cutpoints to define intensity, because slight differences in the low ranges may not be distinguished, such as between sedentary and the lower range of light physical activity. Therefore, the aim of this paper was to explore the performance of the ActiGraph accelerometer in a large population-based surveillance system of youth and adults.

## Methods

Through in-person interviews and physical examinations, the National Health and Nutrition Examination Survey (NHANES) provides a cross-sectional assessment of nutrition and health of the United States population. The data used in this study were obtained between 2003–2006, the most recently available data with accelerometer-assessed physical activity. Participants provided informed consent and children provided assent before completing any questionnaires or measurements. The consenting documents are available for the 2003–2004 (http://www.cdc.gov/nchs/nhanes/nhanes2003-2004/brochures03_04.htm) and 2005–2006 (http://www.cdc.gov/nchs/nhanes/nhanes2005-2006/brochures05_06.htm) cohorts. Our overarching project was reviewed by the University of North Carolina Institutional Review Board and deemed exempt.

Those who participated in the physical activity monitor examination were asked to wear the ActiGraph accelerometer (model #AM7164) on their hip for seven consecutive days during waking hours and outside of any water-based activities. Beginning at midnight on the day following the clinic visit, the accelerometer recorded 1-minute epochs of analog acceleration and converted it to a digital signal [1]. Non-wear was defined by an interval of at least 90 consecutive minutes of zero counts/minute, with allowance of 1 or 2 minutes of nonzero counts if no counts were detected during both the 30 minutes upstream and downstream from that interval; any nonzero counts except the allowed short intervals were considered as wear time [3]. Counts in the non-wear period and values that exceeded 30,000 counts/minute were set to missing [4].

Among youth, we used self-reported or parental reported (for those less than 12 years) sociodemographic measures including age, gender, and race/ethnicity. Measured height and weight were used to derive body mass index, grouped using the year 2000 BMI-for-age growth charts (http://www.cdc.gov/growthcharts/percentile_data_files.htm) as underweight (<5^th^ percentile), healthy weight (5^th^- < 85^th^ percentile), overweight (85^th^- < 95^th^ percentile), and obese (> = 95^th^ percentile). For ages 12 years and older, participants reported their type, frequency, and duration of moderate and vigorous leisure activities over the 30 days preceding the interview. These were summed together in hours/day to derive moderate to vigorous leisure activity.

Among adults, we used self-reported sociodemographic measures including age, gender, race/ethnicity, and education. Adults answered the following question on assistive devices: “Because of a health problem, do you have difficulty walking without using any special equipment?” Measured height and weight were used to derive body mass index, grouped as underweight (<18.5 kg/m^2^), normal weight (18.5- < 25 kg/m^2^), overweight (25- < 30 kg/m^2^), and obese (> = 30 kg/m^2^). Similar to youth ages 12 and older, adult participants were asked to report the type, frequency, and duration of moderate and vigorous leisure activities over the 30 days preceding the interview. These were summed together in hours/day to derive moderate to vigorous leisure activity.

Data for 2003–2006 were combined together for the analyses. Descriptive statistics were calculated to evaluate the performance of the ActiGraph overall and by sociodemographic and health characteristics. P values were generated to compare means using a t-test for 2 level and analysis of variance for > =3 levels of the variable. All analyses were conducted using SAS (version 9.2; Cary, NC).

The youth sample was limited to those ages 6 to 17 years (n = 5,607) who participated in the accelerometer portion of NHANES during 2003–2006 (n = 5030). We further excluded 265 participants whose accelerometer was not in calibration (determined by NHANES upon accelerometer return using PAXCAL variable; 2003–2004: http://wwwn.cdc.gov/nchs/nhanes/2003-2004/PAXRAW_C.htm; 2005–2006: http://www.cdc.gov/nchs/nhanes/nhanes2005-2006/PAXRAW_D.htm), 88 participants whose accelerometer was faulty upon return (i.e., recorded no counts) or deemed not reliable (also determined by NHANES using PAXSTAT variable), and 649 participants who did not provide at least 3 days of accelerometer wear for 8 or more hours per day over the 7-day period. This left a final sample size of 4,028.

The adult sample was limited to those age > =18 years (n = 11,183) who participated in the accelerometer portion of NHANES during 2003–2006 (n = 9,601). We further excluded 450 participants whose accelerometer was not in calibration, 169 participants whose accelerometer was faulty upon return or deemed not reliable, and 1051 participants who did not provide at least 3 days of accelerometer wear for 8 or more hours per day over a 7-day period. This left a final sample size of 7,931.

## Results

Overall the data ranged from 0 to 29,996 counts/minute, with 21,803 different values of counts/minute (72.7% out of 30,001 possible values since we excluded values >30,000 counts/minute). After applying the algorithm to remove non-wearing time, over the one week monitoring period, 27.9% of counts were recorded as zero for youth and 30.1% of counts were recorded as zero for adults. On average, youth had 1381 different counts/minute and adults had 1101 different counts/minute (Table 1). When restricting to counts/minute between 0 to 4999, every possible value (in counts/minute) occurred at least once.

**Table 1:** **Number of different values per person among all counts/minute, overall and by categories for youth and adults; NHANES 2003–2006**

	**Youth 6–17 years (n = 4028)**				**Adults > =18 years (n = 7931)**			
	**Mean (SE)**	**25th percentile**	**50th percentile**	**75th percentile**	**Mean (SE)**	**25th percentile**	**50th percentile**	**75th percentile**
Overall, counts/minute	1381.4 (5.5)	1127.0	1360.0	1623.0	1101.2 (3.7)	874.0	1085.0	1313.0
0 to 99 counts/minute	99.7 (0.0)	100.0	100.0	100.0	99.8 (0.0)	100.0	100.0	100.0
100 to 499 counts/minute	351.2 (0.6)	336.0	361.0	377.0	352.4 (0.5)	338.0	364.0	380.0
500 to 999 counts/minute	305.6 (1.1)	259.0	314.0	360.0	284.0 (1.0)	226.0	296.0	353.0
1000 to 1999 counts/minute	328.0 (1.9)	236.0	320.0	416.0	240.5 (1.6)	136.0	225.0	329.0
2000 to 2999 counts/minute	149.0 (1.2)	94.0	137.0	195.0	75.4 (0.8)	24.0	58.0	107.0
3000 to 3999 counts/minute	72.1 (0.7)	36.0	63.0	100.0	29.8 (0.4)	5.0	16.0	41.0
4000 to 4999 counts/minute	35.3 (0.5)	13.0	27.0	48.0	16.1 (0.3)	2.0	6.0	19.0
> = 5000 counts/minute	42.5 (0.8)	10.0	25.0	56.0	17.4 (0.6)	1.0	4.0	15.0

SE = standard error.

Among youth, the number of different counts was higher among boys, ages 6 to 9 years, Non-Hispanic Blacks, those who were normal weight, and those in the highest tertile of moderate to vigorous leisure activity (Table 2). The number of different counts was also higher for those with a higher number of adherent accelerometer days and on weekdays compared to weekends.

**Table 2:** **Number of different counts across adherent days by sociodemographic and health characteristics among youth 6–17 years (n = 4028); NHANES 2003–2006**

	**Sample size (n)**	**Mean (SE)**	**25th percentile**	**50th percentile**	**75th percentile**	**p value** ^**a**^
Gender:						<.0001
Male	2,026	1453.6 (8.0)	1184.0	1448.0	1701.0	
Female	2,002	1308.4 (7.2)	1075.0	1277.5	1534.0	
Age group:						<.0001
6 to 9	1,049	1621.5 (9.9)	1415.0	1632.0	1835.0	
10 to 14	1,804	1362.5 (7.5)	1130.0	1342.0	1579.0	
15 to 17	1,175	1196.1 (8.5)	992.0	1171.0	1385.0	
Race/ethnicity:						<.0001
Non-Hispanic White	1,019	1351.1 (11.1)	1078.0	1334.0	1621.0	
Non-Hispanic Black	1,352	1412.6 (9.9)	1150.0	1385.5	1647.5	
Hispanic	1,457	1369.7 (8.6)	1129.0	1354.0	1596.0	
Other	200	1410.1 (24.8)	1164.0	1393.0	1646.0	
Body mass index:						<.0001
Under weight (<5th percentile)	114	1394.7 (32.3)	1106.0	1355.0	1656.0	
Normal weight (5th- < 85th percentile)	2,426	1416.6 (7.3)	1152.0	1400.5	1666.0	
Overweight (85th- < 95th percentile)	680	1346.4 (12.8)	1099.0	1318.0	1585.5	
Obese (> = 95th percentile)	792	1303.4 (10.8)	1080.0	1279.0	1509.5	
Missing	16					
Moderate to vigorous leisure activity, self-reported^b^:						<.0001
Tertile 1 (0–2.22 hours)	258	1102.8 (15.7)	928.0	1089.5	1261.0	
Tertile 2 (>2.22-9.68 hours)	255	1190.8 (17.8)	992.0	1171.0	1342.0	
Tertile 3 (>9.68 hours)	262	1250.8 (18.9)	1046.0	1230.5	1448.0	
Missing	3,253					
Number of adherent accelerometer days:						<.0001
3 days	337	979.8 (12.4)	815.0	968.0	1130.0	
4 days	466	1138.6 (11.4)	957.0	1129.0	1296.0	
5 days	702	1285.1 (10.8)	1077.0	1254.0	1475.0	
6 days	1,085	1443.8 (9.3)	1207.0	1430.0	1660.0	
7 days	1,438	1554.2 (8.5)	1323.0	1551.0	1775.0	
Weekends	4,028	424.0 (3.1)	306.0	442.0	562.5	n/a
Weekdays	4,028	957.4 (4.4)	759.5	944.0	1139.0	n/a

n/a = not applicable; SE = standard error.

^a^p value for comparison of the means; uses t-test for 2 level and analysis of variance for > =3 levels.

^b^Asked only for those 12 years and older.

Among adults, the number of different counts was higher among men, ages 35 to 49 years, Hispanics, those with greater than high school education, normal or overweight, did not need special equipment to walk, and in the highest tertile of moderate to vigorous leisure activity (Table 3). Similar to youth, the number of different counts was also higher for those with a higher number of adherent accelerometer days and on weekdays compared to weekends.

**Table 3:** **Number of different counts across adherent days by sociodemographic and health characteristics among adults > =18 years (n = 7931); NHANES 2003-2006**

	**Sample size (n)**	**Mean (SE)**	**25th percentile**	**50th percentile**	**75th percentile**	**p value** ^**a**^
Gender:						<.0001
Men	3,813	1171.4 (5.9)	918.0	1163.0	1412.0	
Women	4,118	1036.2 (4.5)	845.0	1026.0	1226.0	
Age group:						<.0001
18-34	2,530	1167.6 (6.4)	940.0	1141.5	1368.0	
35-49	1,790	1227.2 (7.2)	1015.0	1203.0	1418.0	
50-64	1,627	1126.1 (7.4)	929.0	1119.0	1303.0	
65 and older	1,984	882.4 (6.7)	671.0	862.5	1069.5	
Race/ethnicity:						<.0001
Non-Hispanic White	3,953	1076.5 (5.2)	846.0	1069.0	1298.0	
Non-Hispanic Black	1,727	1078.2 (7.7)	862.0	1062.0	1276.0	
Hispanic	1,927	1178.3 (7.9)	935.0	1158.0	1393.0	
Other	324	1065.7 (16.3)	903.0	1047.5	1222.0	
Education:						<.0001
Less than high school	1,002	1074.7 (12.1)	797.0	1031.0	1325.0	
High school graduate/GED	3,278	1097.1 (5.9)	856.0	1073.0	1311.0	
Greater than high school	3,644	1112.7 (5.2)	908.0	1105.0	1313.0	
Missing	7					
Body mass index (kg/m^2^):						<.0001
Underweight (<18.5)	132	1057.3 (28.5)	821.5	1070.0	1305.0	
Normal (18.5- <25)	2,517	1127.1 (6.9)	889.0	1115.0	1342.0	
Overweight (25- <30)	2,644	1130.5 (6.6)	896.0	1112.0	1350.0	
Obese (> = 30)	2,575	1052.7 (6.0)	841.0	1037.0	1252.0	
Missing	63					
Need special equipment to walk:						<.0001
Yes	660	780.8 (12.1)	535.5	737.0	971.5	
No	6,576	1128.6 (4.0)	905.0	1109.0	1325.5	
Missing	695					
Moderate to vigorous leisure activity, self-reported:						<.0001
Tertile 1 (0–0.93 hours)	2,770	1014.3 (6.7)	762.0	992.0	1236.0	
Tertile 2 (>0.93-4.63 hours)	2,455	1115.3 (6.4)	897.0	1091.0	1313.0	
Tertile 3 (>4.63 hours)	2,688	1178.5 (5.9)	966.5	1167.0	1365.0	
Missing	18					
Number of adherent accelerometer days:						<.0001
3 days	459	819.7 (10.3)	671.0	815.0	949.0	
4 days	645	932.0 (10.7)	768.0	923.0	1069.0	
5 days	998	1059.5 (9.2)	865.0	1048.5	1242.0	
6 days	1,740	1137.5 (7.7)	921.0	1128.5	1340.0	
7 days	4,089	1154.2 (5.4)	923.0	1153.0	1369.0	
Weekends	7,931	356.9 (1.8)	259.0	364.0	463.0	n/a
Weekdays	7,931	744.2 (3.1)	551.0	720.0	904.0	n/a

n/a = not applicable; SE = standard error.

^a^p value for comparison of the means; uses t-test for 2 level and analysis of variance for > =3 levels.

## Discussion

We found that for the NHANES sample, all values between 0 and 4999 counts/minute occurred, spanning the range in which all but the most vigorous activities would occur [5]. For example, among a sample of 5 to 8 year olds, running on the treadmill at 4 mph produced a mean of 4700 counts/minute using a similar model accelerometer [6]. We were unable to confirm continuous options above the vigorous threshold, since time spent in vigorous activity was relatively low for the sample. These findings are in contrast to the performance of the Actical omnidirectional accelerometer, for which the counts/minute were found to not be continuous [2]. Among a sample of 12,750 adults that wore the Actical accelerometer, the mean number of different counts/minute was 112.5 and 49.3% of all potential values were recorded between 0 to 12,000 counts/minute. This is approximately a 10-fold reduction when compared to our mean of 1101 different counts/minute with the ActiGraph accelerometer for NHANES adults. Moreover, when considering the potential range of ActiGraph data to be 0 to 30,000 counts/minute, 72.7% of all possible values occurred with the NHANES sample. While both accelerometers capture sedentary, light, moderate, and vigorous physical activity, the findings indicate that the ActiGraph output is more continuous in nature than the Actical output.

The Actical has lower variability in the sedentary range (such as below 100 counts/minute), indicating that it may more difficult to distinguish whether 0 counts/minute represents sedentary behavior or non-wear when compared to the ActiGraph. Various automated computer algorithms have been developed to remove accelerometer non-wearing time for both the ActiGraph [3,4,7-13] and the Actical [14]. A consensus to define non-wear has not been reached, and our data indicate that the best recommendation may differ between the two accelerometers. Further exploration is warranted to determine the most accurate cleaning algorithms to remove non-wear for both accelerometers. The ActiGraph also offers a low frequency extension option for more recent versions of their accelerometer that may improve cleaning algorithms and be useful among populations for whom the lower intensity range is more important [15].

The variability of the ActiGraph at the low intensity range also indicates that it may better distinguish sedentary from the lower range of light physical activity compared to the Actical, although calibration studies using both accelerometers have not found this to be true for youth [16]. However, a small study of adults found that the ActiGraph GT3X was more sensitive than the Actical to movements in non-vertical planes and at thresholds of <8000 counts/minute (high vigorous activity), but that the Actical was more sensitive above this cutpoint [17].

Similar to the Actical study [2], we found that among adults, the different number of counts/minute were higher among men, younger ages, normal weight, those with higher self-reported moderate to vigorous leisure activity, those who had more adherent accelerometer days, and on weekdays. We also found these findings to be similar among youth. Intuitively it makes sense that those who are more physically active and wear the accelerometer longer would produce more different counts in their accelerometer file.

This study used the ActiGraph AM7164 accelerometer, a uniaxial accelerometer. It is not known whether the data remain continuous when the manufacturer changed the accelerometer in 2005 to contain a micro-electro-mechanical system (MEMS) capacitive accelerometer [15]. It is possible that the data remained consistent, since several studies report comparability across the ActiGraph devices comparing the AM7164 to newer versions [10,18-23]. It is also not known if using different epoch lengths might impact the ActiGraph performance.

## Conclusion

In conclusion, we found that the ActiGraph AM7164 output to be continuous for a wide range of intensity values. The different number of counts was higher among those that were more physically active, particularly since they used more of the moderate and vigorous intensity count options. These findings provide insight into developing more accurate cleaning algorithms to remove periods of non-wear and indicate that these algorithms may need to be specific to the accelerometer being used.

## Abbreviations

NHANES: National health and nutrition examination survey
n/a: not applicable
SE: Standard error
